# A Personalized CYP2C19 Phenotype-Guided Dosing Regimen of Voriconazole Using a Population Pharmacokinetic Analysis

**DOI:** 10.3390/jcm8020227

**Published:** 2019-02-10

**Authors:** Yun Kim, Su-jin Rhee, Wan Beom Park, Kyung-Sang Yu, In-Jin Jang, SeungHwan Lee

**Affiliations:** 1 Department of Clinical Pharmacology and Therapeutics, Seoul National University College of Medicine and Hospital, Seoul 03080, Korea; mn07013@snu.ac.kr (Y.K.); rheesjin@snu.ac.kr (S.-j.R.); ksyu@snu.ac.kr (K.-S.Y.); ijjang@snu.ac.kr (I.-J.J.); 2 Department of Internal Medicine, Seoul National University College of Medicine and Hospital, Seoul 03080, Korea; wbpark1@snu.ac.kr

**Keywords:** antifungal agent, population pharmacokinetics, pharmacogenetics, infectious disease, individualization

## Abstract

Highly variable and non-linear pharmacokinetics of voriconazole are mainly caused by CYP2C19 polymorphisms. This study aimed to develop a mechanistic population pharmacokinetic model including the CYP2C19 phenotype, and to assess the appropriateness of various dosing regimens based on the therapeutic target. A total of 1,828 concentrations from 193 subjects were included in the population pharmacokinetic analysis. A three-compartment model with an inhibition compartment appropriately described the voriconazole pharmacokinetics reflecting auto-inhibition. Voriconazole clearance in the CYP2C19 intermediate metabolizers (IMs) and poor metabolizers (PMs) decreased by 17% and 53% compared to that in the extensive metabolizers (EMs). There was a time-dependent inhibition of clearance to 16.2% of its original value in the CYP2C19 EMs, and the extent of inhibition differed according to the CYP2C19 phenotypes. The proposed CYP2C19 phenotype-guided initial dosing regimens are 400 mg twice daily (bid) for EMs, 200 mg bid for IMs, and 100 mg bid for PMs. This CYP2C19 phenotype-guided initial dosing regimen will provide a rationale for individualizing the optimal voriconazole therapy.

## 1. Introduction

Voriconazole, a derivative of fluconazole, is a broad-spectrum antifungal agent used for the treatment of various fungal infections [1,2]. The mechanism of action of voriconazole is to prevent biosynthesis of ergosterol from lanosterol by inhibiting fungal cytochrome P450 (CYP)-dependent 14α-sterol demethylase, which is an essential step in the synthesis of the cell membrane [1]. Voriconazole has been widely used in clinical settings and administered either intravenously or orally because of its excellent bioavailability (approximately 90%) [3]. However, the clinical use of voriconazole is limited due to adverse events such as visual disturbances, hepatotoxicity, and central nervous system dysfunctions, which are related to high exposure of voriconazole. For this reason, voriconazole requires therapeutic drug monitoring (TDM) in clinical practice [4,5,6].

Voriconazole exhibits highly variable and non-linear pharmacokinetics. This is contributed by many factors, including age, liver function, CYP2C19 phenotype, saturation and auto-inhibition of its own metabolism, and drug-drug interactions (DDIs) [3,7,8,9]. It has been demonstrated that after a single intravenous or oral dose, voriconazole exposure in the CYP2C19 poor metabolizers (PMs) was more than three times that of the extensive metabolizers (EMs) [9]. This supports the fact that the CYP2C19 phenotype is a critical factor responsible for the variability of voriconazole pharmacokinetics. Voriconazole undergoes hepatic metabolism predominantly by CYP2C19 and also by CYP2C9 and CYP3A4 to a lesser extent to form N-oxide as its major metabolite [3]. In vitro studies have shown that the N-oxide metabolite of voriconazole inhibits its own metabolism [10]. It was recently confirmed in humans that both voriconazole and its N-oxide metabolite strongly inhibit CYP3A4 and also CYP2C19 to a lesser extent at the steady-state concentration [11,12]. The extent of this auto-inhibition can vary according to the CYP2C19 genotypes. All these factors constantly add to the difficulty of maintaining voriconazole concentration within the therapeutic range (2.0–5.5 mg/L) [13,14].

Owing to the high inter- and intra-subject variability of voriconazole pharmacokinetics, TDM is recommended by the United States Food and Drug Administration and the Infectious Diseases Society of America [13,15]. With proper use of TDM, voriconazole therapy leads to fewer discontinuations owing to its adverse reactions. In addition to safety, TDM can also improve the efficacy of voriconazole therapy as observed by the number of complete or partial responses (TDM vs. no TDM: 81% vs. 57%) [16]. The current clinical use of TDM for voriconazole is to allow dose adjustments after the first few days of initiation of the standard dosing regimen. However, unexpected adverse reactions and deviations from the therapeutic range can occur at the beginning of the treatment before performing TDM.

Accordingly, the need for development of an initial dosing regimen for voriconazole has been growing. The initial dose selection based on the CYP2C19 phenotype can detect patients at high risk of exposure before the drug administration itself, and can ultimately help to reach optimal therapeutic concentration accurately. The CYP2C19 phenotype-guided dosing regimen is supported by some recent studies, which represent that genotype-directed dosing can help pediatric and renal transplanted patients to timely achieve the therapeutic target concentration [17,18]. However, the current Clinical Pharmacogenetics Implementation Consortium (CPIC^®^) guideline for voriconazole therapy still lacks adequate information on the initial dosing based on the CYP2C19 phenotype [19]. Therefore, this study was performed to develop a population pharmacokinetic model of voriconazole that reflected the influence of intrinsic factors such as the CYP2C19 phenotype, and to assess the appropriateness of various dosing regimens according to the CYP2C19 phenotypes.

## 2. Methods

### 2.1. Study Population

This study included pharmacokinetic data obtained from healthy volunteers and patient populations who participated in five clinical studies with voriconazole conducted at the Seoul National University Hospital (Table S1) [9,16,20,21]. All subjects gave their informed consent for inclusion before they participated in the clinical studies. The clinical studies were conducted in accordance with the Declaration of Helsinki, and the protocols were approved by the institutional review board of Seoul National University Hospital (Seoul, Republic of Korea; Table S1; H-0811-004-261, H-1207-057-417, H-1607-160-779, H-0808-057-254).

The study population in each clinical study (study 1−4: healthy volunteers, and study 5: patients) received intravenous or oral voriconazole, as follows:

- Study 1: a single dose of intravenous voriconazole 200 mg, followed by single and multiple doses of oral voriconazole 200 mg every 12 h [9],
- Study 2: a single dose of oral voriconazole 400 mg [21],
- Study 3: a single dose of intravenous voriconazole 200 mg [20],
- Study 4: a single dose of intravenous voriconazole 200 mg, followed by a single dose of oral voriconazole 200 mg [22],
- Study 5: loading dose of intravenous voriconazole 6 mg/kg or oral voriconazole 400 mg every 12 h on the first day, followed by TDM-based maintenance doses of intravenous voriconazole 4 mg/kg or oral voriconazole 200 mg every 12 h [16].

The CYP2C19 phenotypes were classified as: EMs, intermediate metabolizers (IMs), and PMs based on the CPIC^®^ guideline, as follows: EM, *1/*1; IM, *1/*2, *1/*3, *2/*17; PM, *2/*2, *2/*3, and *3/*3 [19]. One patient identified as a rapid metabolizer (*1/*17) was considered EM for analysis.

### 2.2. Population Pharmacokinetic Analysis

A population pharmacokinetic analysis from logarithmically-transformed concentration data was performed using a non-linear mixed effects modeling approach with NONMEM (version 7.3.0, Icon Development Solutions, Ellicott City, MD, USA). The first-order conditional estimation method with the interaction option was employed to estimate the pharmacokinetic parameters and their variabilities. The population pharmacokinetic model of voriconazole was constructed using the healthy volunteers’ data, which is an intensive sampled data and sequentially developed by incorporating sparsely sampled patients’ data.

The structure model was selected by exploring one-, two-, and three-compartment models with a linear and/or non-linear elimination (i.e., Michaelis-Menten) model. In addition, a hypothetical inhibition compartment was included in the model to describe the non-linear time-dependent pharmacokinetics [23]. The absorption profile of oral voriconazole was described by a first-order process with a lag time, and the absolute bioavailability (F) was estimated using a logit model based on the available pharmacokinetic data. The inter-individual variability for each pharmacokinetic parameter was evaluated using an exponential error model. To describe the residual unexplained variability, three types of residual error models, including additive, proportional, and combined additive and proportional error models were tested for the healthy volunteers’ and the patients’ data independently.

The effect of the potential covariates on the pharmacokinetics of voriconazole was investigated graphically and statistically using a stepwise forward selection and backward deletion approach. The continuous covariates examined were: age, body weight, aspartate aminotransferase (AST), alanine aminotransferase (ALT), serum creatinine, and estimated glomerular filtration rate. These covariates were tested in the model using power functions normalized to their median values or generally accepted typical value (e.g., 70 kg for body weight). The categorical covariates, including sex, CYP2C19 phenotype, liver function abnormality grade according to the Common Terminology Criteria for Adverse Events (version 4.0) [24], and co-medications such as proton pump inhibitors (PPIs such as omeprazole, pantoprazole, and lansoprazole) or glucocorticoids were examined using an exponential function. The CYP2C19 effects were tested using separate categories of IM and PM referenced to EM. A covariate was considered to be statistically significant when the objective function value (OFV) decreased > 3.84 (*p* < 0.05, χ^2^ distribution with 1 degree of freedom) during forward selection, and increased > 6.63 (*p* < 0.01, χ^2^ distribution with 1 degree of freedom) during backward deletion. Only the biologically plausible parameter–covariate relationships were included in the final model.

### 2.3. Model Selection and Validation

Throughout the model development process, model selection was evaluated based on the goodness of fit plots, the estimates, precision of parameters, and the decrease in OFV. The goodness of fit plots consisted of four plots as follows: observations versus population predictions, observations versus individual predictions, conditional weighted residuals versus population predictions, and conditional weighted residuals versus time. The predictive performance of the model for healthy volunteers’ data was assessed graphically by prediction-corrected visual predictive checks (pcVPCs) performed by stratification of the CYP2C19 phenotype, route of drug administration, and dosing frequency. The adequacy of the model was demonstrated by plotting the time course of the observations along with the prediction interval for the simulated values. The predictive performance of the model for the patients’ data was assessed in terms of bias and precision by calculating the numerical estimates of the mean prediction error (MPE in percentage) and the relative root mean squared error (RMSE in percentage), respectively [25]. 

### 2.4. Model-Based Simulation

Based on the final parameter estimates of the developed model, simulations were performed to predict the concentration profiles of voriconazole according to the CYP2C19 phenotypes after multiple oral doses of different dosing regimens. The simulated dosing regimens included the standard oral dose (400 mg every 12 h on the first day followed by 200 mg every 12 h) and various test doses according to the CYP2C19 phenotypes (400 mg every 12 h on the first day followed by 100–400 mg every 12 h). The simulation was done for 7 days, which is considered sufficient to achieve the theoretical steady-state. Using the simulated voriconazole concentration according to the CYP2C19 phenotypes, the probability of attainment of the pharmacokinetic target was calculated, where the target voriconazole trough concentration was predefined as the currently used therapeutic range of 2.0−5.5 mg/L [13,14]. 

## 3. Results

### 3.1. Demographics

A total of 1,828 voriconazole plasma concentration-time data from 93 healthy volunteers (1,579 observations) and 100 patients (249 observations) were included in the population pharmacokinetic analysis (Table 1). Of the total 193 studied population, 164 (85%) were males. The age of the study population ranged from 18 to 80 years, and the body weight ranged from 40.8 to 88.5 kg. The proportions of the CYP2C19 phenotype in EM, IM, and PM were 39% (*n* = 75), 36% (*n* = 70), and 25% (*n* = 48) respectively. 

### 3.2. Population Pharmacokinetic Model

**Figure 1:**
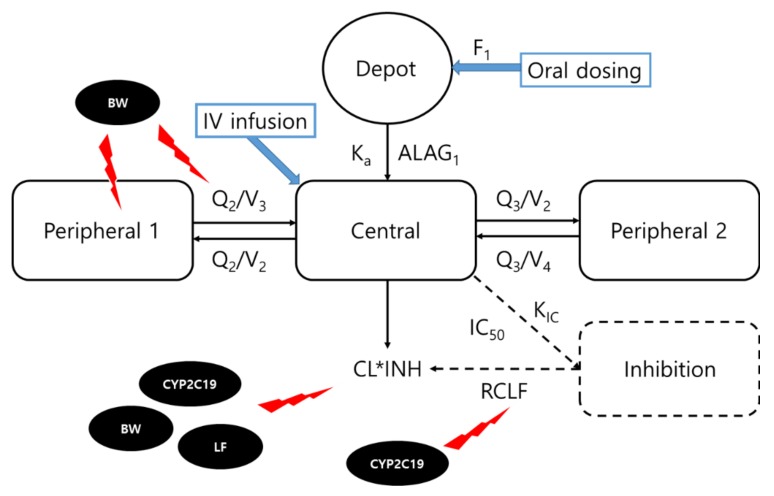
Structure of the population pharmacokinetic model for voriconazole concentrations and significant covariates. Clearance (CL) is inhibited based on the concentration in an empirical inhibition compartment. C_Inh_ corresponds to the concentration in the inhibition compartment. INH corresponds to [RCLF + (1 − RCLF) × (1 − C_Inh_/(IC_50_ + C_Inh_))]; F_1_, bioavailability; K_a_, absorption rate constant; ALAG_1_, absorption lag-time; V_2_, central volume of distribution; Q_2_ and Q_3_, inter-compartmental clearance; V_3_ and V_4_, peripheral volume of distribution; K_IC_, rate constant into inhibition compartment; RCLF, remaining CL fraction, i.e., fraction of clearance which cannot be inhibited; IC_50_, concentration in the inhibition compartment yielding 50% of maximum clearance inhibition; IV, intravenous; BW, body weight; LF, liver function.

A three-compartment model with a first-order oral absorption, an absorption lag time, and elimination along with an inhibition compartment model appropriately described the time-concentration profile of voriconazole, showing distinct non-linear pharmacokinetic behavior (Figure 1). The inhibition compartment reflected the auto-inhibition of voriconazole metabolism, as well as a time-dependent clearance (CL) profile by multiplying the inhibition fraction of voriconazole CL (INH) to the initial CL (CL_0_). The equations for the CL and INH are as follows:

CL = CL_0_ × INH, (1)

(2) $$\mathrm{INH}=\mathrm{RCLF}+\left(1-\mathrm{RCLF}\right)\cdot \left(1-\frac{C_{\mathrm{Inh}}}{{\mathrm{IC}}_{50}+C_{\mathrm{Inh}}}\right)$$

(RCLF is the remaining CL fraction reflecting the CL at a steady state and the fraction that cannot be inhibited, C_Inh_ is the concentration in the inhibition compartment, and IC_50_ is the C_Inh_ yielding 50% of maximum CL inhibition).

Accordingly, the CL is inhibited by the C_Inh_, allowing to select values in the range of 0 to 100% of the CL_0_. Additionally, a rate constant was added to the inhibition compartment (K_IC_) to explain the time course of CL inhibition.

The absorption rate constant of 1.23 h^−1^ and the lag time of 0.237 h appropriately described the absorption phase of orally administered voriconazole. The absolute oral bioavailability of voriconazole was estimated to be 87.6%. The estimated typical CL of voriconazole was 45.3 L/h, which was expected to be inhibited over time up to 16.2% of its original value. Most of the typical parameter values were estimated with a good precision (Table 2). 

In the final model, several covariates that significantly affected the pharmacokinetics of voriconazole were identified (Table 3). The CYP2C19 phenotype significantly affected the pharmacokinetics of voriconazole. The CL of voriconazole decreased by 17% (37.6 L/h) and 53% (21.5 L/h) in the CYP2C19 IMs and PMs respectively compared to that in the CYP2C19 EMs (45.3 L/h). Furthermore, the RCLF also decreased by approximately 36–40% (0.097–0.104) in the CYP2C19 IMs and PMs, compared to that in the CYP2C19 EMs (0.162). Accordingly, the final model accurately predicted the time-dependent CL change, which showed the different magnitude in accordance with the CYP2C19 phenotypes (Figure S1). In addition, body weight was found to be a significant covariate of CL, peripheral volume of distribution (V_3_), and inter-compartmental clearance (Q_2_) of voriconazole. A significant reduction (47%) in voriconazole CL was also observed in patients with liver dysfunction (grade ≥ 3), which indicated that these patients might be at a higher risk of exceeding the target range of voriconazole concentration.

### 3.3. Model Validation

**Figure 2:**
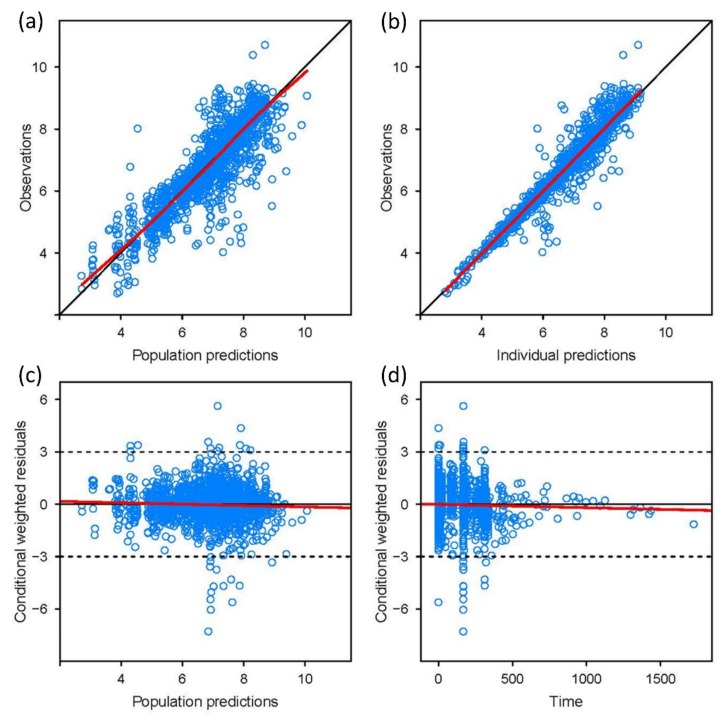
Basic goodness-of-fit plots of final model. (**a**) observations versus population predictions; (**b**) observations versus individual predictions; (**c**) conditional weighted residuals versus population predictions; (**d**) conditional weighted residuals versus time.

The basic goodness of fit (Figure 2) and pcVPC plots (Figure S2) showed a good predictive performance of the developed model and indicated that the model appropriately described the observed voriconazole concentrations in accordance with the CYP2C19 phenotypes and the route of administration of voriconazole. For numerical quantification of the predictive performance for the patient data, the mean bias and precision (MPE and RMSE, respectively) were observed to be well below 25% and remained relatively constant throughout the observations with different CYP2C19 phenotypes (Table S2).

### 3.4. Various Dosing Regimens According to the CYP2C19 Phenotypes

**Figure 3:**
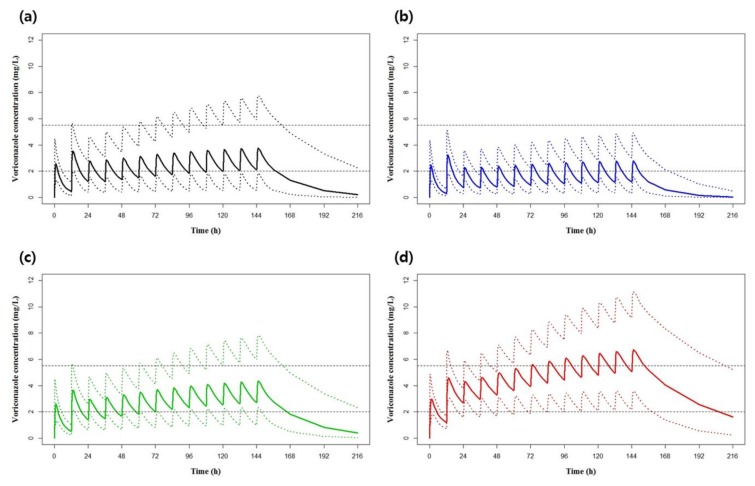
Predicted median concentration–time profile over the first 7 days of treatment of (**a**) total 10,000 simulated patients or patients with (**b**) CYP2C19 EM phenotype, (**c**) IM phenotype, and (**d**) PM phenotype. Standard oral dosing (400 mg twice daily for two doses followed by 200 mg twice daily) was used. The solid lines represent the median, with the dotted lines representing the 10th and 90th percentiles. The dashed lines represent the therapeutic target range for voriconazole trough plasma concentration of 2.0 to 5.5 mg/L.

**Figure 4:**
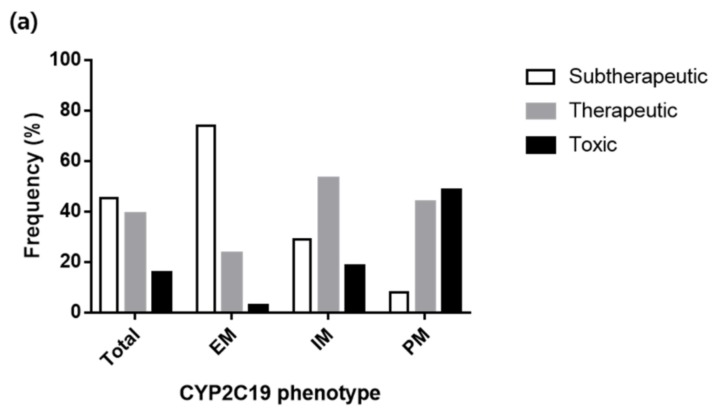

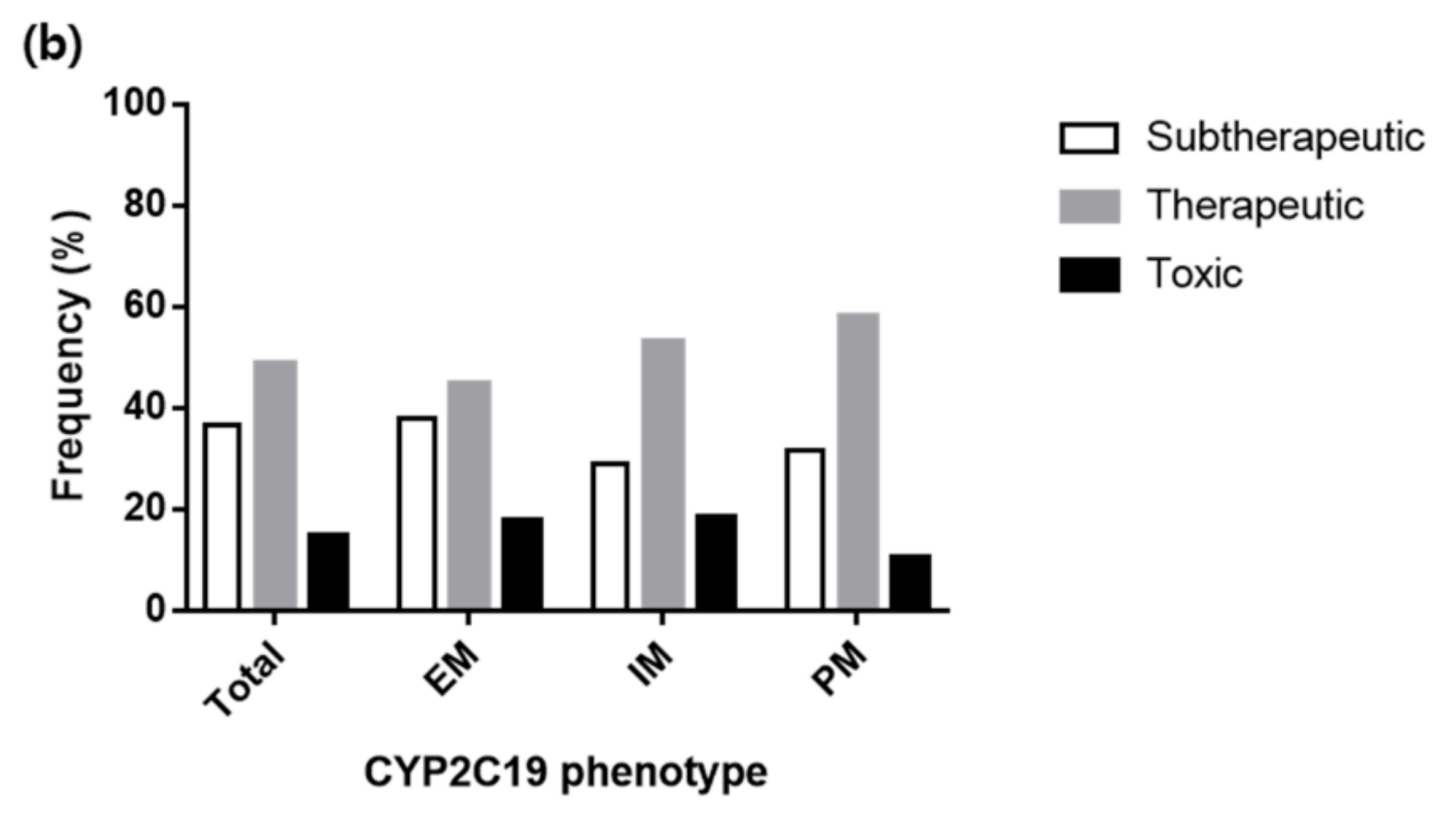
Probability of voriconazole therapeutic target attainment from model-based simulations of voriconazole pharmacokinetic profiles after the following voriconazole oral dosing regimens on day 7; (**a**) standard dosing regimen (400 mg twice daily for two doses followed by 200 mg twice daily), (**b**) dosing according to CYP2C19 phenotype as follows: EM, 400 mg twice daily; IM, 400 mg twice daily for two doses followed by 200 mg twice daily; PM, 400 mg twice daily for two doses followed by 100 mg twice daily. Therapeutic target range for voriconazole trough plasma concentration was from 2.0 to 5.5 mg/L.

Based on the final population pharmacokinetic model, the concentration-time profiles of voriconazole after 7-day multiple oral doses of standard dosing regimen (400 mg twice daily for two doses followed by 200 mg twice daily) were simulated according to the CYP2C19 phenotypes (Figure 3). On average, the trough concentrations after 7-day dosing seemed to reach within the target trough range of 2.0−5.5 mg/L. However, when classified according to the CYP2C19 phenotypes, the trough concentrations reached mostly below the target range in the subjects with EM, while the trough concentrations in the subjects with PM were higher than the target range. Likewise, the evaluation of the probability of voriconazole therapeutic target attainment by the standard oral dosing regimen showed that only 38.9% of the subjects’ concentration fell within the therapeutic target range. The probabilities of subtherapeutic concentration attainment were high (73.9%) in the subjects with EM, while the subjects with PM showed high toxic concentration attainment (48.3%), suggesting the need for dose adjustment according to the CYP2C19 phenotypes (Figure 4a, Table S3). 

To find the appropriate dosing regimen for each CYP2C19 phenotype, we evaluated the target attainments for the various oral dosing regimens (Table S3) and suggested the dosing regimens to achieve the highest probability of reaching the therapeutic concentration as follows: EM: 400 mg twice daily, IM: 400 mg twice daily for two doses followed by 200 mg twice daily, and PM: 400 mg twice daily for two doses followed by 100 mg twice daily. The optimal dosing regimen resulted in higher probabilities of therapeutic target attainment in each CYP2C19 phenotype (i.e., 44.7%, 52.9%, and 58.1% for EM, IM, and PM respectively) compared to the standard dosing regimen. In addition, the probability of subtherapeutic concentration attainment in the subjects with EM and the probability of toxic concentration attainment in the subjects with PM was significantly decreased by the suggested CYP2C19 phenotype-guided dosing (Figure 4b, Table S3).

## 4. Discussion

The present study was performed to develop a population pharmacokinetic model of voriconazole, taking into account the important covariates, including the CYP2C19 phenotype, and to evaluate the appropriateness of various dosing regimens according to the CYP2C19 phenotypes. This study is scientifically meaningful because the population pharmacokinetic analysis includes a sufficient number of subjects whose CYP2C19 phenotypes were all identified, and there was a sufficient number of subjects with intensive pharmacokinetic sampling. This study is also worthwhile, as the developed model is the first mechanistic model incorporating the auto-inhibitory characteristic of voriconazole to illustrate its non-linear pharmacokinetic characteristics with time-dependent elimination. Through this study, we quantitatively identified the effects of the CYP2C19 phenotype on voriconazole pharmacokinetics and suggested an initial dosing regimen based on that. 

The developed mechanism-based model better explain the non-linear pharmacokinetic profile of voriconazole than the previous models. So far, the elimination of voriconazole has been described as a linear [26,27], non-linear [28,29,30], or mixed (linear and non-linear) [31,32] process. The underlying mechanism of the non-linear pharmacokinetic property of voriconazole is supported by its CYP-mediated auto-inhibition and saturation of its own metabolism [12,33]. In addition, richly sampled pharmacokinetic data from the healthy volunteers helped to develop a robust model and overcome the disturbances from sparse data obtained from the patients. Therefore, it was possible to determine the accurate elimination profile of voriconazole that reflects the mechanistic auto-inhibition properly.

Among the various covariates affecting the pharmacokinetics of voriconazole, the CYP2C19 phenotype, a major factor that contributes to the high variability of voriconazole exposure was identified as a significant covariate of both CL and RCLF. In the present study, the CL of voriconazole decreased by 17% (37.6 L/h) and 53% (21.5 L/h) in the CYP2C19 IMs and PMs respectively compared to that in the EMs (45.3 L/h). This result was relatively consistent with some previous studies showing 37% reduction of the linear CL in CYP2C19 PMs [27], and approximately 40% reductions of the maximum rate of metabolism (V_max_) in non-linear (Michaelis-Menten) kinetics in CYP2C19 IMs/PMs or PMs [29,32]. 

The RCLF of voriconazole was adopted to the model to illustrate its non-linear pharmacokinetic characteristics with time-dependent elimination by its auto-inhibitory characteristic. In this study, the time-dependent CL of voriconazole was found to be different according to the CYP2C19 phenotypes. The RCLF decreased by approximately 36−40% (0.097−0.104) in the CYP2C19 IMs or PMs, compared to that in the EMs (0.162). This signifies that when the steady-state is reached, the time-dependent CL changes from 45.3 to 7.3 L/h in CYP2C19 EMs, from 37.6 to 3.9 L/h in IMs, and from 21.5 to 2.1 L/h in PMs respectively (Table 2, Table 3 and Figure S1). Although there has not been any report with the IMs and PMs, the result with the EMs was consistent with a previous study which reported approximately 80% reduction of V_max_ at steady state in CYP2C19 UMs/EMs [31]. The time-dependent inhibition characteristic is a key factor to understand the non-linear pharmacokinetics of voriconazole. Therefore, it is important to consider not only the simple change in CL but also the change in CL over time according to the CYP2C19 phenotypes. To the best of our knowledge, the present study is the first to quantitatively identify the effect of each CYP2C19 phenotype on CL, as well as on RCLF. 

In addition to the CYP2C19 phenotype, several other covariates were identified which need to be considered during the clinical use of voriconazole. Among them, liver dysfunction significantly affected the CL of voriconazole, demonstrating up to 47% reduction of CL in patients with grade ≥ 3 of liver dysfunction (Table 3). This result can be supported by the recommended maintenance dose in patients with mild to moderate hepatic insufficiency (Child-Pugh Class A and B), which is halved due to 3.2-fold higher mean AUC of voriconazole than that in controls with normal hepatic function [33]. It was also reported by some previous studies that severe hepatic cholestasis significantly lowers the voriconazole CL by approximately 10%, and the CL was reduced by 16% as alkaline phosphatase level increased by 100 (U/L) [26,27]. However, further evaluation is needed due to limited data available from only 6 patients with grade ≥ 3 hepatic abnormality. In addition, body weight was also identified as a significant covariate of the CL, V_3_, and Q_2_, although the influence of body weight was not sufficient to change the dosing regimen. It is inconsistent with the prescribing information of voriconazole, which recommends that the oral maintenance dose should be 100 or 150 mg for adult patients weighing less than 40 kg [33]. This might occur because there was no patient with a body weight of less than 40 kg in our study. 

In the present study, none of the co-medications (PPIs and glucocorticoids) were identified as a significant covariate on voriconazole exposure. In contrast, recent studies have reported the exposure of voriconazole increased to varying degrees depending on the kinds of PPIs used [34,35], although, the role of glucocorticoids on voriconazole exposure remains controversial [18,29,35,36,37]. This inconsistency may be because of the small number of patients who had taken those concomitant medications in this study, yielding sparse pharmacokinetic data. Although the effects of PPIs and glucocorticoids on voriconazole exposure have not been yet confirmed, attention should be granted by clinicians since this combination is used commonly.

The standard dosing regimen of voriconazole has been suggested as an oral dose of 400 mg bid for two doses followed by 200 mg bid for adults regardless of the CYP2C19 phenotypes [33]. The proposed CYP2C19 phenotype-guided oral dosing regimen to maximize the probability of timely achieving the therapeutic range of 2.0−5.5 mg/L is 400 mg bid for two doses followed by 400 mg bid in the EMs, 200 mg bid in the IMs, or 100 mg bid in the PMs. The suggested dose appears to be slightly higher than the conventional dose because the target trough range is higher than the conventional range (0.5−5.5 mg/L) [38,39]. Another proposed regimen that maximizes the probability of achieving the therapeutic concentration while keeping attainment of the probability of toxic concentration under 20% is as follows: 400 mg bid for two doses, followed by 300–400 mg bid for the EMs, 400 mg bid for two doses followed by 150–200 mg bid for the IMs, and 400 mg bid for two doses followed by 100 mg bid for the PMs (Table S3). Although we could not evaluate the potential effect of *17 on voriconazole exposure due to only one patient having *17, there is a need for further study to confirm since a previous report showed that higher doses of voriconazole are needed to maximize the benefit for CYP2C19 rapid metabolizer (*1/*17) and ultrarapid metabolizer (*17/*17) [40]. In the clinical setting, one of the variously proposed dosing regimens can be selected based on the patient’s condition, taking into account the likelihood of therapeutic success and the risk of adverse effects. 

Despite the proposed CYP2C19 phenotype-guided oral dosing regimen, challenges still remain in real life situations [14]. There are difficulties in obtaining the CYP2C19 genotype of the patients before initiating voriconazole therapy due to the time and cost associated with the laboratory process. In addition, a strong consensus for therapeutic range and dosing regimen of voriconazole has not been reached yet. These limitations can be compensated by the CYP2C19 phenotype-guided dosing in patients with CYP2C19 phenotype and followed by TDM-guided dose adjustments. 

## 5. Conclusions

In conclusion, the pharmacokinetic parameters of voriconazole were well described by the developed population pharmacokinetic model. This was the first attempt to mechanistically explain the non-linear pharmacokinetics of voriconazole using an inhibition compartment model. The proposed CYP2C19-guided initial dosing regimen based on the final model will provide a rationale to individualize optimal dosing to improve clinical outcomes with voriconazole therapy.

## Figures and Tables

**Table 1:** Demographic and clinical characteristics of study population.

Variables	Total (*n* = 193)	Healthy Subjects ^a^ (*n* = 93)	Patients ^b^ (*n* = 100)
Age (years)	34 (18–80)	26 (20–41)	59 (18–80)
Weight (kg)	66.0 (40.8–88.5)	70.3 (57.6–88.5)	59.4 (40.8–86.4)
Aspartate aminotransferase (U/L)	21 (7–377)	18 (9–40)	30 (7–377)
Alanine aminotransferase (U/L)	21 (4–363)	15 (4–52)	29 (4–363)
Sex			
Male	164 (85)	93 (100)	71 (71)
Female	29 (15)	-	29 (29)
CYP2C19 phenotype			
Extensive metabolizer	75 (39)	32 (34)	43 (43)
Intermediate metabolizer	70 (36)	27 (29)	43 (43)
Poor metabolizer	48 (25)	34 (37)	14 (14)
Liver function abnormality ^c^			
Grade 0	165 (85.5)	93 (100)	72 (72)
Grade 1	9 (4.7)	-	9 (9)
Grade 2	13 (6.7)	-	13 (13)
Grade 3	5 (2.6)	-	5 (5)
Grade 4	1 (0.5)	-	1 (1)
Co-medication			
Proton pump inhibitors	22 (11.4)	-	22 (22)
Steroids	9 (4.7)	-	9 (9)

Data were presented as number of subjects (%) except for age, weight, aspartate aminotransferase, and alanine aminotransferase which were presented as median (range). ^a^ Study 1–4; ^b^ Study 5; ^c^ Liver function abnormality according to Common Terminology Criteria for Adverse Events (version 4.0).

**Table 2:** Parameter estimates of the final population pharmacokinetic model.

Parameters	Estimates	RSE (%)
**Structural model**		
V_2_; central volume of distribution (L)	35.7	15.7
CL; clearance (L/h)	45.3	5.8
V_3_; peripheral 1 volume of distribution (L)	58.9	6.2
Q_2_; inter-compartmental clearance between central and peripheral 1 compartment (L/h)	10.9	8.0
V_4_; peripheral 2 volume of distribution (L)	25.4	16.7
Q_3_; inter-compartmental clearance between central and peripheral 2 compartment (L/h)	54.6	45.4
K_a_; absorption rate constant (h^−1^)	1.23	15.4
F_1_; bioavailability	0.876	2.3
ALAG_1_; absorption lag-time (h)	0.237	1.8
RCLF; fraction of clearance which cannot be inhibited	0.162	9.7
IC_50_; concentration in the inhibition compartment yielding 50% of maximum clearance inhibition	0.01 FIX	NA
K_IC_; rate constant into inhibition compartment	0.002	14.9
**Inter-individual variability (IIV)**		
IIV for V_2_ (% CV)	40.2	23.3 ^a^
IIV for CL (% CV)	21.4	10.6 ^a^
IIV for V_3_ (% CV)	20.6	34.1 ^a^
IIV for Q_2_ (% CV)	28.8	20.0 ^a^
IIV for K_a_ (% CV)	87.8	14.4 ^a^
IIV for F_1_ (% CV)	84.4	20.3 ^a^
IIV for RCLF (% CV)	54.4	13.0 ^a^
Correlation between V_2_ and CL	0.0116	95.7 ^b^
Correlation between V_2_ and V_3_	−0.0117	200.9 ^b^
Correlation between V_2_ and Q_2_	−0.0734	49.2 ^b^
Correlation between CL and V_3_	−0.0119	72.5 ^b^
Correlation between CL and Q_2_	0.008	150.3 ^b^
Correlation between V_3_ and Q_2_	0.0345	67.0 ^b^
**Residual variability**		
Additive error for healthy subjects (mg/L)	0.208	8.4
Additive error for patients (mg/L)	0.799	6.7

RSE, relative standard error; NA, not applicable; ^a^ Standard error given on the variance scale; ^b^ Standard error of the covariance estimate.

**Table 3:** Significant covariate effects on the population pharmacokinetic parameters in the final model.

Variable	Estimates	RSE (%)
**Effect on CL**		
Body weight exponent for CL	0.595	31.8
CYP2C19 phenotype effect for CL (cf. 0 for extensive metabolizer)		
Intermediate metabolizer	−0.186 ^a^	29.5 ^a^
Poor metabolizer	−0.746 ^a^	10.9 ^a^
Liver function abnormality effect for CL (cf. 0 for grade < 3)		
Grade ≥ 3	−0.75	49.3
**Effect on V_3_**		
Body weight exponent for V_3_	2.2	20.0
**Effect on Q_2_**		
Body weight exponent for Q_2_	2.56	18.1
**Effect on RCLF**		
CYP2C19 phenotype effect for CL (cf. 0 for extensive metabolizer)		
Intermediate metabolizer	−0.51 ^a^	27.5 ^a^
Poor metabolizer	−0.44 ^a^	42.3 ^a^

RSE, relative standard error; CL, clearance; V_3_, peripheral 1 volume of distribution; Q_2_, inter-compartmental clearance between central and peripheral 1 compartment; RCLF, fraction of clearance which cannot be inhibited; ^a^ The values were estimated using healthy subject data.

## Supplementary Materials

The following are available online at http://www.mdpi.com/2077-0383/8/2/227/s1, Table S1. Detailed information on the pharmacokinetic data of each clinical study; Table S2. Predictive performance of voriconazole pharmacokinetic model for the data from the patients; Table S3. Probabilities of target attainment on day 7 from model-based simulations of voriconazole pharmacokinetic profiles after 400 mg twice daily for two doses followed by various voriconazole oral dosing regimens; Figure S1. Predicted voriconazole clearance versus time after oral dosing of 400 mg twice daily for two doses followed by 200 mg twice daily; Figure S2. Prediction-corrected visual predictive check for healthy subject data in the final pharmacokinetic model. The circles represent the observed concentrations. The lines represent the median (red) and the 5th and 95th percentiles (blue) of the observed concentration. The areas represent the 95% confidence intervals for the median (red) and 90% prediction interval (blue) of the simulated concentrations.
