# Supplementary material for: A Personalized CYP2C19 Phenotype-Guided Dosing Regimen of Voriconazole Using a Population Pharmacokinetic Analysis
Source: J Clin Med. 2019 Feb 10;8(2):227. doi: 10.3390/jcm8020227 (PMC6406770; doi:10.3390/jcm8020227)

Supplementary Figure S1. Predicted voriconazole clearance versus time after oral dosing of 400 mg twice daily for two doses followed by 200 mg twice daily

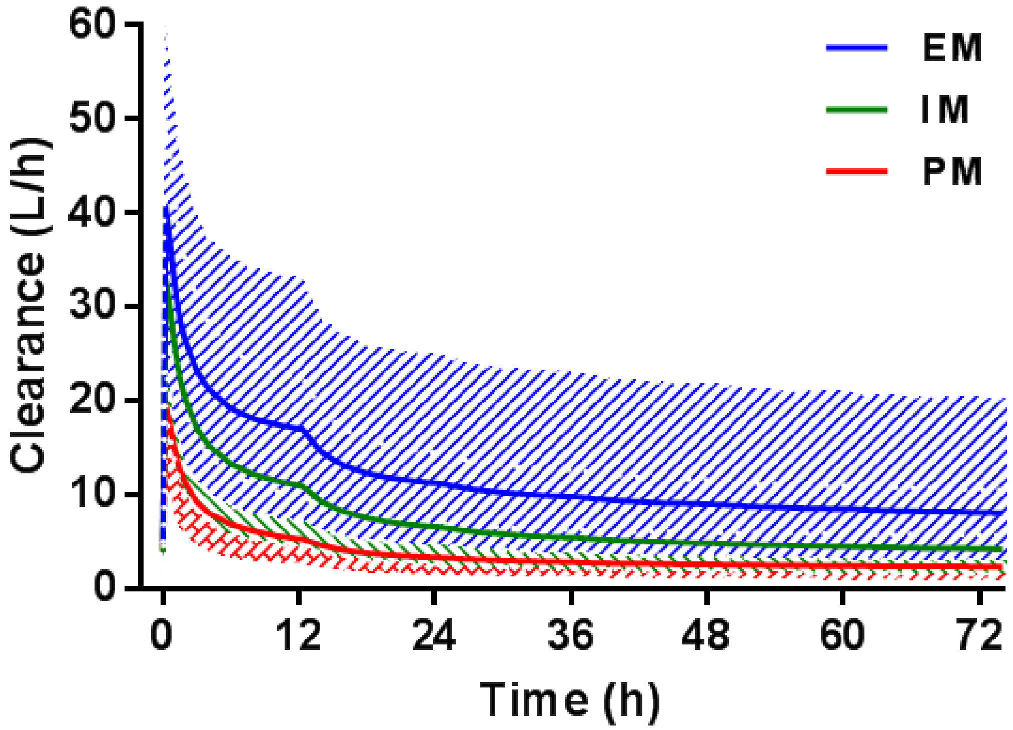

Supplement: Supplementary file 1 [file jcm-08-00227-s001.zip › Supplementary Figure S1.pdf]
