# Supplementary material for: A Personalized CYP2C19 Phenotype-Guided Dosing Regimen of Voriconazole Using a Population Pharmacokinetic Analysis
Source: J Clin Med. 2019 Feb 10;8(2):227. doi: 10.3390/jcm8020227 (PMC6406770; doi:10.3390/jcm8020227)
Supplement: Supplementary file 1 [file jcm-08-00227-s001.zip › Supplementary Figure S2.pdf]

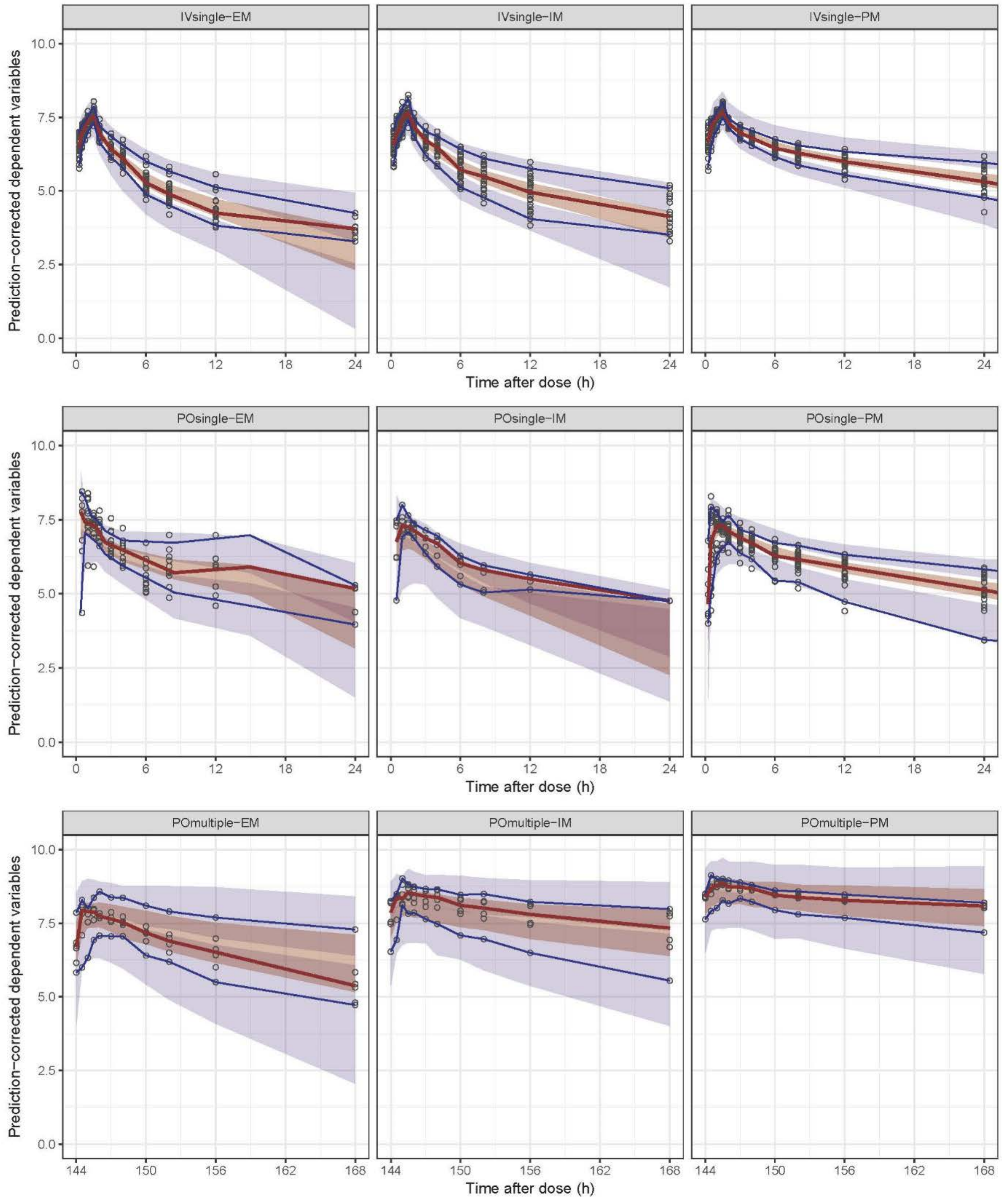

Supplementary Figure S2. Prediction-corrected visual predictive check for healthy subject data in the final pharmacokinetic model. The circles represent the observed concentrations. The lines represent the median (red) and the 5th and 95th percentiles (blue) of the observed concentration. The areas represent the 95% confidence intervals for the median (red) and 90% prediction interval (blue) of the simulated concentrations.
