# Supplementary material for: A Personalized CYP2C19 Phenotype-Guided Dosing Regimen of Voriconazole Using a Population Pharmacokinetic Analysis
Source: J Clin Med. 2019 Feb 10;8(2):227. doi: 10.3390/jcm8020227 (PMC6406770; doi:10.3390/jcm8020227)
Supplement: Supplementary file 1 [file jcm-08-00227-s001.zip › Supplementary Table S1.docx]

# Supplementary Table S1. Detailed information on the pharmacokinetic data of each clinical study.

|  | | **Clinical studies** | | | | |
| --- | --- | --- | --- | --- | --- | --- |
|  | **Study 1 [1]** | | **Study 2 [2]** | **Study 3 [3]** | **Study 4**  **(unpublished data)** | **Study 5 [4]** |
| Population | Healthy subjects | | Healthy subjects | Healthy subjects | Healthy subjects | Patients |
| Number of subjects | 18 | | 12 | 51 | 12 | 100 |
| Treatments | Single 200 mg IV (day 1);  and single 200 mg PO (day 8) followed by multiple 200 mg PO bid (day 9-14) | | Single 400 mg PO | Single 200 mg IV | Single 200 mg IV (day 1); and single 200 mg PO (day 8) | Loading doses of 6 mg/kg IV or 400 mg PO bid (day 1), followed by TDM based maintenance dose |
| PK sampling time | - **Single IV on day 1**   - pre-dose, 0.25, 0.5, 1, 1.5, 2, 3, 4, 6, 8, 12, 24 h post-dose (total 12 points) - **Single PO on day 8**   - pre-dose, 0.5, 1, 1.5, 2, 3, 4, 6, 8, 12, 24 h post-dose (total 11 points) - **Multiple PO on day 9-14**   - trough levels on day 12 and 13; and pre-dose, 0.5, 1, 1.5, 2, 3, 4, 6, 8, 12, 24 h post-dose on day 14 (total 14 points) | | - **Single PO on day 1**   - pre-dose, 0.5, 1, 1.5, 2, 3, 4, 6, 8, 12, 24 h post-dose (total 11 points) | - **Single IV on day 1**   - pre-dose, 0.25, 0.5, 0.75, 1, 1.5, 2, 3, 4, 6, 8, 12, 24 h post-dose (total 13 points) | - **Single IV on day 1**   - pre-dose, 0.25, 0.5, 1, 1.5, 2, 3, 4, 6, 8, 12, 24, 48, 72 h post-dose (total 14 points) - **Single PO on day 8**   - pre-dose, 0.25, 0.5, 1, 1.5, 2, 3, 4, 6, 8, 12, 24, 48, 72 h post-dose (total 14 points) | - **IV or PO after day 4**   - trough levels 4 days after treatment initiation (total 249 points) |
| IRB number | H-0811-004-261 | | | H-1207-057-417 | H-1607-160-779 | H-0808-057-254 |
| NCT number | NCT00942773 | | NCT01080651 | NCT01657201 | NCT02906176 | NCT00890708 |

IV, intravenous; PO, oral; bid, twice daily; TDM, therapeutic drug monitoring

# References

1. Lee S, Kim BH, Nam WS, et al. Effect of CYP2C19 polymorphism on the pharmacokinetics of voriconazole after single and multiple doses in healthy volunteers. J Clin Pharmacol **2012**; 52(2): 195-203.

2. 이승환. Inductive effect of rifampin on the clinical pharmacokinetics of voriconazole and related biomarkers / 이승환. 서울: 서울 : 서울대학교 대학원, **2012**.

3. Chung H, Lee H, Han HK, et al. A pharmacokinetic comparison of two voriconazole formulations and the effect of CYP2C19 polymorphism on their pharmacokinetic profiles. Drug Des Devel Ther **2015**; 9: 2609-16.

4. Park WB, Kim NH, Kim KH, et al. The effect of therapeutic drug monitoring on safety and efficacy of voriconazole in invasive fungal infections: a randomized controlled trial. Clin Infect Dis **2012**; 55(8): 1080-7.
