# Supplementary material for: A Personalized CYP2C19 Phenotype-Guided Dosing Regimen of Voriconazole Using a Population Pharmacokinetic Analysis
Source: J Clin Med. 2019 Feb 10;8(2):227. doi: 10.3390/jcm8020227 (PMC6406770; doi:10.3390/jcm8020227)
Supplement: Supplementary file 1 [file jcm-08-00227-s001.zip › Supplementary Table S2.docx]

# Supplementary Table S2. Predictive performance of voriconazole pharmacokinetic model for the data from the patients

|  | N^a^ | Bias and imprecision | |
| --- | --- | --- | --- |
|  |  | MRE (%)^b^ | RMSE (%)^c^ |
| Total | 249 | 2.0 (-0.6, 4.6) | 20.7 |
| CYP2C19 EM | 113 | -2.8 (-6.8, 1.3) | 21.9 |
| CYP2C19 IM | 104 | 4.6 (0.8, 8.5) | 20.4 |
| CYP2C19 PM | 32 | 10.3 (5.5, 15.2) | 17.2 |
| ^a^ N represents the number of observations ^b^ Data in parentheses are 95 % confidence intervals; MPE, mean prediction error;  ^c^ RMSE, relative root mean squared error | | | |
