# Supplementary material for: A Personalized CYP2C19 Phenotype-Guided Dosing Regimen of Voriconazole Using a Population Pharmacokinetic Analysis
Source: J Clin Med. 2019 Feb 10;8(2):227. doi: 10.3390/jcm8020227 (PMC6406770; doi:10.3390/jcm8020227)
Supplement: Supplementary file 1 [file jcm-08-00227-s001.zip › Supplementary Table S3.docx]

# Supplementary Table S3. Probabilities of target attainment on day 7 from model-based simulations of voriconazole pharmacokinetic profiles after 400 mg twice daily for two doses followed by various voriconazole oral dosing regimens.

| CYP2C19 Phenotype | Target attainment | 100 mg BID | 150 mg BID | 200 mg BID | 250 mg BID | 300 mg BID | 350 mg BID | 400 mg BID |
| --- | --- | --- | --- | --- | --- | --- | --- | --- |
| EM | Subtherapeutic | 95.1 | 86.1 | 73.9 | 61.9 | 51.9 | 45.6 | 37.8 |
|  | Therapeutic | 4.8 | 13.0 | 23.3 | 32.8 | 39.0 | 41.6 | 44.7 |
|  | Toxic | 0.1 | 0.9 | 2.8 | 5.3 | 9.1 | 12.9 | 17.5 |
| IM | Subtherapeutic | 69.3 | 46.0 | 28.8 | 18.7 | 13.9 | 10.3 | 6.2 |
|  | Therapeutic | 28.9 | 46.7 | 52.9 | 51.1 | 46.2 | 38.6 | 35.5 |
|  | Toxic | 1.8 | 7.3 | 18.3 | 30.2 | 39.9 | 51.1 | 58.3 |
| PM | Subtherapeutic | 31.6 | 14.9 | 8.0 | 3.8 | 2.4 | 1.4 | 0.8 |
|  | Therapeutic | 58.1 | 57.1 | 43.7 | 32.9 | 24.6 | 18.4 | 13.3 |
|  | Toxic | 10.3 | 28.0 | 48.3 | 63.4 | 73.0 | 80.2 | 85.9 |
| Total | Subtherapeutic | 75.1 | 58.9 | 45.3 | 35.2 | 28.6 | 24.2 | 19.0 |
|  | Therapeutic | 22.6 | 33.7 | 38.9 | 40.7 | 40.1 | 37.1 | 36.3 |
|  | Toxic | 2.3 | 7.4 | 15.8 | 24.1 | 31.3 | 38.8 | 44.6 |
| BID, twice daily |  |  |  |  |  |  |  |  |
